# Evaluation of cardiac autonomic dysfunctions in children with type 1 diabetes mellitus

**DOI:** 10.1186/s12887-024-04644-y

**Published:** 2024-04-01

**Authors:** Davut Gözüküçük, Berkut A. İleri, Serra Karaca Başkan, Ece Öztarhan, Dilek Güller, Hasan Önal, Kazım Öztarhan

**Affiliations:** 1 Department of Medicine, Division of Pediatrics, Sağlık Bilimleri University, Kanuni Sultan Süleyman Training and Research Hospital, Atakent Mh, Turgut Özal Bulvari No:46/1, Küçükçekmece, 34303 Istanbul, Turkey; 2 Department of Medicine, T.C. Demiroğlu Bilim University İstanbul Florence Nightingale Hospital, İzzetpaşa Mah, Abide-I Hürriyet Cd No:166, Şişli, 34381 Istanbul, Turkey; 3 Department of Medicine, Division of Pediatrics, Subdivision of Pediatric Cardiology, Istanbul University, Istanbul Faculty of Medicine Training and Research Hospital, Turgut Özal Millet St., Istanbul, Fatih, Topkapı 34093 Turkey; 4 Department of Medicine, Yeditepe University, Yeditepe Faculty of Medicine Training and Research Hospital, Koşuyolu, Koşuyolu Cd. No: 168, Kadıköy, 34718 Istanbul, Turkey; 5 Department of Medicine, Division of Pediatrics, Subdivision of Pediatric Gastroenterology, T.C. Demiroğlu Bilim University, İstanbul Florence Nightingale Hospital, İzzetpaşa Mah, Abide-I Hürriyet Cd No:166, Şişli, 34381 Istanbul, Turkey; 6 Department of Medicine, Division of Pediatrics, Subdivision of Pediatric Endocrinology and Metabolism, Sağlık Bilimleri University, Başakşehir Çam ve Sakura City Hosptial, Başakşehir Mahallesi G-434 Caddesi No: 2L, Başakşehir, Istanbul, Turkey

**Keywords:** Tissue Doppler, Tilt table test, Heart rate variability, Cardiac autonomic dysfunction, Type 1 diabetes mellitus

## Abstract

**Background:**

Cardiovascular autonomic neuropathy (CAN) is a serious complication of diabetes, impacting the autonomic nerves that regulate the heart and blood vessels. Timely recognition and treatment of CAN are crucial in averting the onset of cardiovascular complications. Both clinically apparent autonomic neuropathy and subclinical autonomic neuropathy, particularly CAN pose a significant risk of morbidity and mortality in children with type 1 diabetes mellitus (T1DM). Notably, CAN can progress silently before manifesting clinically. In our study, we assessed patients with poor metabolic control, without symptoms, following the ISPAD 2022 guideline. The objective is is to determine which parameters we can use to diagnose CAN in the subclinical period.

**Methods:**

Our study is a cross-sectional case–control study that includes 30 children diagnosed with T1DM exhibiting poor metabolic control (average HbA1c > 8.5% for at least 1 year) according to the ISPAD 2022 Consensus Guide. These patients, who are under the care of the pediatric diabetes clinic, underwent evaluation through four noninvasive autonomic tests: echocardiography, 24-h Holter ECG for heart rate variability (HRV), cardiopulmonary exercise test, and tilt table test.

**Results:**

The average age of the patients was 13.73 ± 1.96 years, the average diabetes duration was 8 ± 3.66 years, and the 1-year average HbA1c value was 11.34 ± 21%. In our asymptomatic and poorly metabolically controlled patient group, we found a decrease in HRV values, the presence of postural hypotension with the tilt table test, and a decrease in ventricular diastolic functions that are consistent with the presence of CAN. Despite CAN, the systolic functions of the ventricles were preserved, and the dimensions of the cardiac chambers and cardiopulmonary exercise test were normal.

**Conclusions:**

CAN is a common complication of T1DM, often associated with the patient’s age and poor glycemic control. HRV, active orthostatic tests, and the evaluation of diastolic dysfunctions play significant roles in the comprehensive assessment of CAN. These diagnostic measures are valuable tools in identifying autonomic dysfunction at an early stage, allowing for timely intervention and management to mitigate the impact of cardiovascular complications associated with T1DM.

## Background

Type 1 Diabetes Mellitus (T1DM) is a prevalent chronic disorder affecting children and adolescents. Clinical symptoms typically peak between 5 and 7 years old and during early puberty. These peaks are attributed to increased infection rates during school ages, elevated sex steroids, growth hormone levels, and heightened psychological stress in adolescents [1, 2].

Complications of T1DM can be broadly categorized as microvascular (e.g., peripheral neuropathies, autonomic neuropathies, retinopathy, nephropathy) and macrovascular (e.g., coronary heart disease, cerebrovascular disease, peripheral vascular disease) [3].

Autonomic neuropathies in adult patients have been identified as significant contributors to diabetes-related mortality, leading to dysregulations in cardiovascular, gastrointestinal, and genitourinary functions, pupillary responses, sweat gland activity, and regulatory responses against hypoglycemia. Cardiovascular autonomic neuropathy, an important yet lesser-known complication of diabetes mellitus, is associated with nearly doubling mortality rates [4]. Studies have established a correlation between autonomic function disorders and factors such as age, prolonged diabetes duration, and poorly maintained metabolic control, with increased prevalence in patients exhibiting poor glycemic control [5].

Recognizing disruption in baroreceptor susceptibility is crucial for identifying autonomic function disorders in T1DM patients [6]. A decrease in baroreceptor susceptibility heightens sympathetic nervous system excitability, potentially leading to tachycardia by affecting the sinoatrial node [7, 8]. The presence of cardiac autonomic neuropathy (CAN) is linked with arterial stiffness in both adult and young diabetes patients. Factors contributing to decreased baroreceptor susceptibility include endothelial dysfunction [9, 10], oxidative stress [11, 12], the Rho/Rho Kinase pathway [13], arginase mechanism, and adhesion molecules involved in initiating sympatho-sympathetic feedback reflexes [14, 15].

Controversy exists regarding the early detection of subclinical signs of autonomic dysfunction in children with diabetes [16–18]. However, not only clinically apparent autonomic neuropathy but also subclinical autonomic neuropathy, particularly cardiac autonomic neuropathy (CAN), pose a significant risk of morbidity and mortality in children with T1DM. Some suggest that CAN may progress silently over time before becoming clinically manifest [1, 19]. It has been estimated that diabetic patients with CAN have a 3.4 times higher risk of mortality than those without CAN.Recognition and treatment of autonomic cardiac functions not only decrease cardiovascular damage but may also decrease the disease’s mortality and morbidity rates with proper breathing and exercise education [13, 20].

Our study aimed to recognize CAN early in the subclinical period in patients with poor metabolic control. As per the ISPAD 2022 consensus guideline, the categorization of metabolic control is defined as follows: good metabolic control (%HbA1c < 7.5%), moderate metabolic control (%HbA1c 7.5–8.5%), and poor metabolic control (HbA1c% > 8.5%) [21]. who are without clinical symptoms of CAN. We examined the parameters necessary for the early diagnosis of cardiovascular dysfunctions that may develop due to cardiac autonomic dysfunctions in pediatric patients with T1DM.

## Methods

Our study is a cross-sectional case–control investigation involving 30 children diagnosed with Type 1 Diabetes Mellitus (T1DM) exhibiting poor metabolic control (HbA1c% > 8.5%), as per the ISPAD 2022 Consensus Guide, at the pediatric endocrinology clinic of the University of Health Sciences. Comprehensive medical, endocrinological, cardiological, and neurological histories were obtained, examined, and meticulously recorded for all participants.

Relevant diabetes-related factors, including the duration of diabetes, insulin dosage, glycemic control, and instances of hypoglycemic events, were extracted from medical records. Adhering to the ISPAD 2022 guideline [21], well-controlled T1DM was defined by an HbA1c < 7%, whereas poor metabolic control was indicated by HbA1c > 8.5%. To ensure a representative measure of poor glycemic control, HbA1c values over a 1-year period were averaged.

In our study, the inclusion criteria for the study group are as follows: a minimum 1-year average HbA1c level > 8.5, patients aged between 5–18, and the ability to comply with and complete all the tests. For the control group, we selected healthy children with similar demographic characteristics to the study group, devoid of any additional diseases, and capable of adapting to and completing all the tests.

Excluded from the study were children with associated issues known to influence the outcomes of cardiac autonomic function, such as medical diseases (e.g., heart failure), medications impacting heart rate or rhythm (e.g., beta-blockers, digitalis, theophylline, thyroid hormones, tricyclic antidepressants, anti-arrhythmic drugs, atropine, and its derivatives), symptoms suggesting cardiac arrhythmia documented by electrocardiography (ECG) recording, history of febrile illness in the past week, conditions with symptoms mimicking autonomic neuropathy but not true autonomic neuropathy (e.g., syncope), presence of ketoacidosis or hypoglycemia during the study period, clinically manifest autonomic neuropathy, and children with test results that have suboptimal precision.

All children in both control and study groups underwent evaluation by the same cardiologist physician, who had no prior information about the patients. Traditional echocardiographic measurements were conducted using a 3.5–5 MHz transducer device (General ElectricTM Vivid-5S model), incorporating M mode, CW Doppler, PW Doppler, and Doppler Tissue Imaging mods. Video-recorded samples were analyzed, and to mitigate the impact of heart rate on diastolic functions, 7 cycle samples were collected, and the arithmetic mean was calculated. Systolic and diastolic functions of the ventricles were assessed through cardiac measurements using M mode, PW Doppler, and Doppler Tissue Imaging. Myocardial Performance Index (MPI) was calculated for both ventricles separately, obtained by dividing the sum of isovolumetric contraction time (IVCT) and isovolumetric relaxation time (IVRT) by ventricular contraction time ejection time (VCT) [22]. Conventional echocardiographic methods included measuring E wave, A wave, E/A wave ratio, and deceleration time with mitral valve PW Doppler. Tissue Doppler was employed to measure ejection time, relaxation time, contraction time from the septum, and myocardial systolic and diastolic waves from the apical four chambers and the lateral wall.

Left Ventricular Mass Index (LVMI), expressed in grams per square meter (g/m^2^), was used to normalize left ventricular mass to body surface area. LVMI values greater than 115 g/m^2^ in men and greater than 95 g/m^2^ in women were indicative of Left Ventricular Hypertrophy (LVH). Relative Wall Thickness (RWT) was calculated by dividing the sum of septal and posterior wall thicknesses by the left ventricular internal diameter at end-diastole (LVIDd). The formula for calculating RWT is: RWT = (Septal wall thickness + Posterior wall thickness) / LVIDd. Relative Wall Thickness (RWT) assesses left ventricular remodeling, with a normal RWT typically considered to be less than 0,42 [23–25].

MPI, unaffected by heart rate, ventricular structure, and afterload, is a Doppler index evaluating systolic and diastolic functions together. This index, previously demonstrated to increase in diabetic patients and be effective in revealing diastolic dysfunction, was calculated by dividing the sum of isovolumetric relaxation time (ICZ) and isovolumetric relaxation time (IVRT) by ejection time (ET), as suggested by Tei et al. [26].

24-h rhythm Holter ECG recordings were obtained from all patients using a DMS-300 Holter recording device (DMS, Nevada, USA). Recordings were analyzed by the same cardiologist physician utilizing the DMS Cardioscan model 10 analyzer system (DMS, Nevada, USA). Various heart rate parameters, including 24-h mean heart rate, ectopic beats, presence of block, Standard Deviations of all NN intervals (SDNN), mean of the standard deviations of all NN intervals for all 5-min segments of the entire recording (SDNNI), the standard deviation of the averages of NN intervals in all 5-min segments of the entire recording (SDANNI), the square root of the mean of the sum of the squares of differences between adjacent NN intervals (rMSSD), the number of pairs of adjacent NN intervals differing by more than 50 ms divided by the total number of all NN intervals (pNN50), total power (TPow), very low-frequency range power (VLF power), high-frequency range power (HF power), and low-frequency range power (LF power), were recorded. This analysis aimed to determine the relationship between heart rate changes (using time parameters) and poor glycemic index and durations of diabetes [27].

All patients underwent evaluation through a cardiopulmonary exercise test (CPET) using a treadmill device model Mortara. The cardiopulmonary exercise tests were conducted following the Bruce protocols. Twelve-lead electrocardiographs were recorded both at the initiation and during the procedures. Blood pressure levels were monitored during exercise at 3-min intervals and at the 0th, 5th, 10th, and 30th minutes of the resting period. The duration of exercise, maximum systolic blood pressure (SBP), diastolic blood pressure (DBP) during exercise, and maximum apex beat were recorded. Test termination criteria were established, including ST depressions equal to or more than 2 mm compared to the starting electrocardiography, ST segment elevations equal to or more than 2 mm compared to the starting electrocardiography, systolic blood pressure lowering by more than 10%, onset of bradycardia, systolic blood pressure elevation to more than 210 mmHg in males and 180 mmHg in females, onset of class 3–4 angina, onset of severe arrhythmias, reaching the targeted heart rate, and feeling overwhelmed to the extent of being unable to continue testing. This part of our study aims to determine the relationship between effort capacity, effort duration, poor glycemic index, and the duration of diabetes.

All patients were evaluated by a tilt table test. Patients fasted for 4 to 6 h before the test. The procedure was conducted with a tilt-adjustable table. Patients lay down on the table when it was in a horizontal state, and they remained in this position for 5 min before starting the test. Vascular access was established, and patients were monitored to track heart rate and blood pressure values. After the test started, patients waited for 20 to 45 min on a 60 to 70-degree angled table; this phase of the test is referred to as the passive phase. If no syncope developed, patients proceeded to the second phase. In the second phase, 300 μg sublingual nitroglycerine was applied, and patients waited at the table, under the same conditions as the passive phase, for 15 to 20 min. The entire process lasted about 45 to 85 min (5 min for lying down, 20 to 45 min for the passive phase, and 15 to 20 min for the second phase). Test termination criteria were established as the onset of syncope (accompanied by arrhythmias and/or hypotension) or the patient not wanting to continue testing. Rates of developing orthostatic hypotension, syncope, and presyncope in patients were recorded. The definition of postural hypotension was determined as SBP decreasing by equal to or more than 20 mmHg or DBP decreasing equal to or more than 10 mmHg during the test compared to the start of the test. This part of our study aims to determine the relationship between sympathetic vasoreflexes, poor glycemic index, and the duration of diabetes.

The recorded data were analyzed using the program “IBM Corp. Released 2016. IBM SPSS Statistics for Macintosh, Version 24.0. Armonk, NY: IBM Corp.” The normal distribution of the data was tested through both visual (Q-Q, Box Plot, Stem, and Leaf, and histogram graphs) and analytical (Shapiro–Wilk and Kolmogorov–Smirnov tests) methods. Descriptive statistics were presented using Mean ± standard deviation (SD) for data showing normal distribution graphs (parametric), and median (lowest-highest value) for data not showing normal distribution graphs (non-parametric). Non-parametric data between groups were evaluated using Mann Whitney U and Kruskal Wallis tests, while parametric data between groups were assessed using the student’s T test. Categorical data were compared using Chi-square and Fisher Exact tests. Spearman and Pearson correlation analysis tests were employed to evaluate the relationship between metric data. The confidence interval was set at 95%, and cases where the p-value was below 0.05 were considered statistically significant.

## Results

Patients diagnosed with T1DM in the pediatric cardiology outpatient clinic and individuals in the healthy control group underwent comprehensive assessments based on height, weight, and BMI. No statistically significant differences were identified between the patient and control groups concerning age, weight, height, and body surface area, as indicated in Table 1.

**Table 1:** Comparison of the age and body measurements between both groups

**Variables**	**Group A**	**Group B**	***P* value**
	**(*n* = 30)**	**(*n* = 30)**	
**Age (year)**	13,46 (± 1,96)^c^	11,46 (± 3,04)^c^	0,34^a^
**Weight (kg)**	53,5 (25—73)^d^	39 (18—72)^d^	0,030^b^
**Height (cm)**	156,1 (± 10,8)^c^	147,2 (± 14,4)^c^	**0,012**^**a**^
**BMI (kg/m**^**2**^**)**	21,95 (± 3,65)^c^	18,77 (13,15—27,1)^d^	**0,02**^**b**^
**Weight (Z-Score)**	2.36^e^	2.18^e^	0.52^f^
**Height (Z-Score)**	2.34^e^	2.88^e^	0.44^f^
**BMI (Z-Score)**	1.86^e^	1.45^e^	0.57^f^

Group A: Study group. Group B: Control group

*BMI* Body mass index

X^a^: Student’s T test has been used for parametric tests

X^b^: Mann–Whitney U test has been used for non-parametric tests

X^c^: “mean” and “standard deviation” values have been indicated in parenthesis for parameters that suit normal distribution

X^e^: Standard deviation calculations has been used for Z-score tests

X^f^: Z-test has been used for non-parametric tests

X^d^: “median” and “minimum–maximum” values have been indicated in parenthesis for parameters that does not suit normal distribution

Thirty patients (female/male: 18/12) were followed up with a diagnosis of type 1 diabetes mellitus, with an average age of 13.73 ± 1.96 (10–17) for the patient group. For the control group, data from a total of 60 healthy individuals with an average age of 11.46 ± 3.04 (8–12) (girls/boys: 16/14) were evaluated. The average duration of diabetes in the patient group was 8 ± 3.66 (1 to 16) years. The 1-year average of HbA1C values for our patients was 11.34% ± 2.14% (8.5% to 16.4%).

In our investigation, echocardiographic M-mode examinations disclosed an increase in left ventricular end-systolic diameter (LVDs), left ventricular diastolic diameter (LVDd), left ventricular mass (LVM), and left ventricular mass index (LVMI). Nevertheless, this increase did not achieve statistical significance. Notably, BMI was significantly higher in the patient group, and echocardiographic findings aligned with the elevation in BMI. In Group A, there were noteworthy increments in pulmonary artery late diastolic flow velocity (PA) and pulmonary artery late diastolic flow time (PAT), indicative of right ventricular diastolic dysfunction. This increase was statistically significant when compared to the control group, as depicted in Table 2.

**Table 2:** Comparison of the echocardiographic M-mode measurements between both groups

**Variables**	**Group A**	**Group B**	***P* value**
	**(*n* = 30)**	**(*n* = 30)**	
**IVSd (cm)**	0,6 (0,4—0,8)^d^	0,6 (0,4—0,8)^d^	0,311^b^
**IVSs (cm)**	1 (0,7—1,4)^d^	0,9 (0,6—1,5)^d^	0,296^b^
**LVDd (cm)**	4,4 (3,3—5)^d^	3,9 (3,5—4,9)^d^	0,168^b^
**LVDs (cm)**	2,5 (1,6—3,2)^d^	2,3 (1,6—4,6)^d^	0,114^b^
**LVPWd (cm)**	0,7 (0,5—1)^d^	0,6 (0,5—2,5)^d^	0,107^b^
**LVPWs (cm)**	1,5 (1,1—1,9)^d^	1,35 (0,6—1,8)^d^	0,248^b^
**EDV (mm**^**3**^**)**	87 (43—118)^d^	67 (51—110)^d^	0,189^b^
**ESV (mm**^**3**^**)**	21,5 (8—42)^d^	19 (8—36)^d^	0,052^b^
**EF (%)**	73,5 (63—85)^d^	74 (66—87)^d^	0,149^b^
**SF (%)**	61 (34—96)^d^	65 (37—72)^d^	0,22^b^
**LA (cm)**	2,88 (± 0,38)^c^	2,66 (± 0,41)^c^	0,067^**a**^
**Ao (cm)**	2,32 (± 0,34)^c^	2,28 (± 0,37)^c^	0,865^**a**^
**LA/Ao (unitless)**	1,21 (1—1,71)^d^	1,26 (1,06—1,5)^d^	0,362^b^
**LVMI (g/m**^**2**^**)**	55,32 (± 12,5)	50,7 (19,96—291,86)	0,811
**LVM (g)**	83,52 (± 24,02)	61,61 (35,8—329,82)	0,078
**RWT (cm)**	0,34 (0,23–10,43)^d^	0,32 (0,26—1,09)^d^	0,491^b^
**PA (m/s)**	0,25 (0,04—0,47)^d^	0,2 (0,18—0,26)^d^	** < 0,001**^**b**^
**PAT (ms)**	44 (30—64)^d^	34,6 (30—44)^d^	** < 0,001**^**b**^

Group A: Study group. Group B: Control group

*IVSd* End diastolic intraventricular septum thickness, *IVSs* End systolic intraventricular septum thickness, *LVDd* Left ventricle end-diastolic diameter, *LVDs* Left ventricle end-systolic diameter, *LVPWd* End diastolic left ventricle posterior wall thickness, *LVPWs* End systolic left ventricle posterior wall thickness, *EDV* End diastolic ventricle volume, *ESV* End systolic ventricle volume, *EF* Ejection fraction, *SF* Shortening fraction, *LA* Left atrium width, *Ao* Aortic root, *LA/Ao* Left atrium width ratio to the aortic root, *LVMI* Left ventricle mass index, *LVM* Left ventricle mass, *RWT* Relative wall thickness, *PA* Pulmonary artery late diastolic flow velocity, *PAT* Pulmonary artery late diastolic flow time

X^a^: Student’s T test has been used for parametric tests

X^b^: Mann–Whitney U test has been used for non-parametric tests

X^c^: “mean” and “standard deviation” values have been indicated in parenthesis for parameters that suit normal distribution

X^d^: “median” and “minimum–maximum” values have been indicated in parenthesis for parameters that does not suit normal distribution

When assessing right ventricular systolic and diastolic functions using tissue Doppler, a reduction in E_RV_, E/A_RV_, VCT_RV_, and TD_RV_, indicative of diastolic dysfunction, was observed. Additionally, an elevation in A_RV_ was noted, and these changes were statistically significant. However, no statistically significant differences were found in the evaluation of E/E’_RV_, VC_RV_, IVCT_RV_, IVRT_RV_, ET_RV_, AT_RV_, VC_RV_, IVCT_RV_, IVRT_RV_, DECT_RV_, and PHT_RV_. The study revealed a statistically significant difference in the average right ventricular myocardial performance index (MPI-RV) values between the patient group (0.27, range: 0.21–0.65) and the healthy group (0.23, range: 0.16–0.28) (*p* < 0.001). Details are provided in Table 3.

When assessing left ventricular systolic and diastolic functions using Tissue Doppler, no statistically significant difference was observed in E_LV_, E/A_LV_, E/E’_LV_, VCT_LV_, TD_LV_, A_LV_, VC_LV_, ET_LV_, AT_LV_, IVCT_LV_, and IVRT_LV_. However, there was an increase in IVCT_LV_ values and a decrease in VCT_LV_, IVRT_LV_, TD_LV_, DECT_LV_, and PHT_LV_, and these changes were found to be statistically significant. The study revealed a statistically significant difference in the average left ventricular myocardial performance index (MPI-LV) values between the patient group (0.26, range: 0.18–0.55) and the healthy group (0.2, range: 0.16–0.3) (*p* < 0.001). Please refer to Table 3 for comprehensive details.

In the study, 24-h Holter electrocardiography measurements exhibited statistically significant differences between groups in variables such as 24-h mean heart rate (MHR), standard deviations of all NN intervals (SDNN), mean of the standard deviations of all NN intervals for all 5-min segments of the entire recording (SDNNI), the standard deviation of the averages of NN intervals in all 5-min segments of the entire recording (SDANNI), rMSSD, pNN50, total power (TPow), very low-frequency range power (VLF power), high-frequency range power (HF power), and low-frequency range power (LF power). Refer to Table 4 for comprehensive data.Upon evaluating data from cardiopulmonary exercise test (CPET) measurements, it was determined that the maximum systolic blood pressure was significantly higher during exercise, and this increase achieved statistical significance compared to the control group. However, no significant differences were found between the groups concerning maximum exercise capacity, maximum heart rate, and maximum systolic blood pressure values, as detailed in Table 5.

In our study, data collected from tilt table tests revealed statistically significant differences (*p* = 0.024) in the rates of developing orthostatic hypotension, syncope, and presyncope between groups, as presented in Table 6.

**Table 3:** Comparison of the Doppler Tissue Imaging measurements of the right and the left ventricle between both groups

**Variables**	**Group A**	**Group B**	***P* value**
	**(*n* = 30)**	**(*n* = 30)**	
**E**_**RV**_** (m/s)**	0,14 (0,09—0,19)^d^	0,16 (0,11—0,19)^d^	**0,050**^**b**^
**E**_**LV**_** (m/s)**	0,15 (0,12—0,21)^d^	0,17 (0,11—0,21)^d^	0,333^b^
**A**_**RV**_** (m/s)**	0,14 (0,06—0,21)^d^	0,08 (0,07—0,23)^d^	** < 0,001**^**b**^
**A**_**LV**_** (m/s)**	0,06 (0,04—0,09)^d^	0,07 (0,03—0,08)^d^	0,796^b^
**E/A**_**RV**_** (unitless)**	1,05 (0,64—2,33)^d^	1,8 (1,2—2,71)^d^	** < 0,001**^**b**^
**E/A**_**LV**_** (unitless)**	2,5 (1,6—4,75)^d^	2,7 (0,68—3,3)^d^	0,784^b^
**E/E’RV (unitless)**	4,59 (0—6,55)^d^	4,19 (3,16—7,64)^d^	0,717^b^
**E/E’LV (unitless)**	5,81 (± 1,12)^c^	6,2 (± 1,33)^c^	0,227^a^
**VC**_**RV**_** (m/s)**	0,13 (0,11—0,17)^d^	0,13 (0,1—0,17)^d^	0,058^b^
**VC**_**LV**_** (m/s)**	0,1 (0,07—0,16)^d^	0,09 (0,06—0,15)^d^	0,446^b^
**IVCT**_**RV**_** (m/s)**	0,09 (0,05—0,18)^d^	0,09 (0,06—0,13)^d^	0,152^b^
**IVCT**_**LV**_** (m/s)**	0,05 (0,02—0,09)^d^	0,05 (0,03—0,11)^d^	0,208^b^
**IVRT**_**RV**_** (m/s)**	0,05 (0,03—0,08)^d^	0,04 (0,03—0,05)^d^	0,676^b^
**IVRT**_**LV**_** (m/s)**	0,04 (0,03—0,06)^d^	0,04 (0,03—0,07)^d^	**0,031**^**b**^
**ET**_**RV**_** (ms)**	135,75 (72,5—196,3)^d^	130,25 (83,5—166)^d^	0,695^b^
**ET**_**LV**_** (ms)**	98,6 (64,3—122)^d^	103,6 (82,4—124,7)^d^	0,093^b^
**AT**_**RV**_** (ms)**	95,4 (74,3—120,6)^d^	86,2 (41,2—125,7)^d^	0,424^b^
**AT**_**LV**_** (ms)**	60 (40,3—92,7)^d^	63,35 (49,5—118,3)^d^	0,195^b^
**VCT**_**RV**_** (ms)**	219,9 (171,5—258,7)^d^	247,6 (217,8—303,3)^d^	** < 0,001**^**b**^
**VCT**_**LV**_** (ms)**	231,15 (105,5—289)^d^	246,7 (222—309,2)^d^	**0,005**^**b**^
**IVCTT**_**RV**_** (ms)**	36,15 (33—67)^d^	34 (25,6—44)^d^	**0,041**^**b**^
**IVCTT**_**LV**_** (ms)**	33 (18,5—61)^d^	26,55 (22—44)^d^	** < 0,001**^**b**^
**IVRTT**_**RV**_** (ms)**	22 (15—33)^d^	22 (22—33)^d^	0,102^b^
**IVRTT**_**LV**_** (ms)**	22 (22—27,5)^d^	22 (22—27,5)^d^	0,175^b^
**TD**_**RV**_** (ms)**	314,3 (249,5—521,3)^d^	387,95 (266—628,3)^d^	**0,017**^**b**^
**TD**_**LV**_** (ms)**	314,65 (239,6—510,3)^d^	372,5 (266—617,6)^d^	**0,012**^**b**^
**TS**_**RV**_** (ms)**	294,4 (248,6—429,4)^d^	299 (188,3—338,5)^d^	0,679^b^
**TS**_**LV**_** (ms)**	302,23 (± 36,98)^c^	308,39 (± 18,16)^c^	0,287^a^
**DECT**_**RV**_** (ms)**	101,82 (± 24,04)^c^	96,04 (± 24,84)^c^	0,624^a^
**DECT**_**LV**_** (ms)**	67,49 (± 10,96)^c^	58,19 (± 10,97)^c^	**0,003**^**a**^
**PHT**_**RV**_** (ms)**	30,16 (± 6,74)^c^	27,55 (± 6,45)^c^	0,251^a^
**PHT**_**LV**_** (ms)**	20 (15—26,5)^d^	17,5 (11,5—22,4)^d^	**0,023**^**b**^
**MPI**_**RV**_** (unitless)**	0,27 (0,21—0,65)^d^	0,23 (± 0,03)^c^	** < 0,001**^**b**^
**MPI**_**LV**_** (unitless)**	0,26 (0,18—0,55)^d^	0,2 (0,16—0,3)^d^	** < 0,001**^**b**^

Group A: Study group. Group B: Control group

*E* E wave velocity, *A* A wave velocity, *E/A* E wave velocity to A wave velocity ratio, *E/E’RV* E value found in Tricuspitt PW Doppler to E value found in RV Tissue Doppler Imaging ratio, *E/E’LV* E value found in Mitral PW Doppler to E value found in LV Tissue Doppler Imaging ratio, *VC* Ventricular contraction velocity, *IVCT* Isovolumetric contraction velocity, *IVRT* Isovolumetric relaxation velocity, *ET* E wave time, *AT* A wave time, *VCT* Ventricular contraction time, *IVCTT*: Isovolumetric contraction time, *IVRTT* Isovolumetric relaxation time, *TD* Total diastole time, *TS* Total systole time, *DECT* Deceleration time, *PHT* Pressure half time, *MPI* Myocardial performance index

X_RV_: Measurements of the right ventricle

X_LV_: Measurements of the left ventricle

X^a^: Student’s T test has been used for parametric tests

X^b^: Mann–Whitney U test has been used for non-parametric tests

X^c^: “mean” and “standard deviation” values have been indicated in parenthesis for parameters that suit normal distribution

X^d^: “median” and “minimum–maximum” values have been indicated in parenthesis for parameters that does not suit normal distribution

**Table 4:** Comparison of the 24-h Holter ECG measurements between both groups

**Variables**	**Group A**	**Group B**	***P* value**
	**(*n* = 30)**	**(*n* = 30)**	
**MAX QT**_**C**_** (ms)**	485 (320—692)^d^	510 (473—812)^d^	0,063^b^
**SDNN (ms)**	115 (51—169)^d^	148,3 (± 26,27)^c^	** < 0,001**^**b**^
**SDNNI (unitless)**	48,7 (± 9,75)^c^	68,9 (± 13,36)^c^	** < 0,001**^**a**^
**SDANNI**	104 (43—149)^d^	128,2 (93—193)^d^	** < 0,001**^**b**^
**rMSSD (ms)**	26 (14—60)^d^	43,1 (± 12,58)^c^	** < 0,001**^**b**^
**pNN50 (%)**	6 (0—32)^d^	18,1 (± 9,43)^c^	** < 0,001**^**b**^
**TPow (Hz)**	2418,8 (652,7—5713)^d^	4771,4 (1626—8538,5)^d^	** < 0,001**^**b**^
**VLF power (Hz)**	1529,9 (370,7—3780)^d^	2999,2 (858—5569,8)^d^	** < 0,001**^**b**^
**LF power (Hz)**	531,2 (190,4—1054)^d^	1095,31 (± 401,3)^c^	** < 0,001**^**b**^
**HF power (Hz)**	348,6 (81,8—931,3)^d^	632,1 (121,6—1487,9)^d^	**0,001**^**b**^
**LF/HF (unitless)**	2,1 (0,8—7,7)^d^	2,5 (0,5—9,9)^d^	0,080^b^
**MHR (bpm)**	92,1 (± 7,79)^c^	81,2 (± 6,99)^c^	** < 0,001**^**a**^

Group A: Study group. Group B: Control group

*SDNN* The standard deviation of all NN intervals, *SDNNI* The mean of the standard deviations of all NN intervals for all 5-min segments of the entire recording, *SDANNI* The standard deviation of the averages of NN intervals in all 5-min segments of the entire recording, *rMSDD* The square root of the mean of the sum of the squares of differences between adjacent NN intervals, *pNN50* The number of pairs of adjacent NN intervals differing by more than 50 ms divided by the total number of all NN intervals, *TPow* Total power, *VLF power* The very low-frequency range power, *LF power* The low-frequency range power, *HF power* The high-frequency range power, *MHR* 24-h mean heart rate

X^a^: Student’s T test has been used for parametric tests

X^b^: Mann–Whitney U test has been used for non-parametric tests

X^c^: “mean” and “standard deviation” values have been indicated in parenthesis for parameters that suit normal distribution

X^d^: “median” and “minimum–maximum” values have been indicated in parenthesis for parameters that does not suit normal distribution

**Table 5:** Comparison of the cardiopulmonary exercise tests (CPET) between both groups

**Variables**	**Group A**	**Group B**	***P* value**
	**(*n* = 30)**	**(*n* = 30)**	
**EC**_**MAX**_** (Minutes)**	12,4 (4,6—13,4)^c^	12,85 (7—14,9)^c^	0,544^a^
**HR**_**MAX**_** (bpm)**	189 (111—250)^c^	186,07 (± 8,45)^b^	0,127^a^
**Exercise duration (Minutes)**	12,12 (± 2,43)^b^	12,42 (9,34—18,33)^c^	0,496^a^
**SBP**_**MAX**_** (mmHg)**	155,53 (± 17,91)^b^	143 (104—208)^c^	**0,026**^**a**^
**DBP**_**MAX**_** (mmHg)**	79,5 (52—145)^c^	76 (51—116)^c^	0,728^a^

Group A: Study group. Group B: Control group

*EC*_*MAX*_ Maximum exercise capacity, *HR*_*MAX*_ Maximum heart rate, *SBP*_*MAX*_ Maximum systolic blood pressure, *DBP*_*MAX*_ Maximum diastolic blood pressure

X^a^: Student’s T test has been used for parametric tests

X^b^: Mann–Whitney U test has been used for non-parametric tests

X^c^: “mean” and “standard deviation” values have been indicated in parenthesis for parameters that suit normal distribution

X^d^: “median” and “minimum–maximum” values have been indicated in parenthesis for parameters that does not suit normal distribution

**Table 6:** Comparison of the tilt table test results between both groups

**Tilt table test**	**Study Group (*n* = 30)**	**Control Group (*n* = 30)**	***P* value**
**Negative**	24 (%80)	30 (%100)	**0,024**^**a**^
**Positive**	6 (%20)	0 (%0)	

X^a^: Fisher exact test has been used

## Discussion

Diabetic autonomic neuropathy (DAN) is a significant complication of T1DM. Until the last two decades, DAN was often overlooked, and its prevalence was underestimated. It was commonly perceived as a rare and/or late complication of diabetes [1]. DAN is characterized by dysfunction or damage to the parasympathetic and/or sympathetic branches of the autonomic nervous system (ANS) in individuals with diabetes, following the exclusion of other potential causes of autonomic neuropathy [28]. Clinical manifestations of DAN vary depending on the affected organ and can include symptoms related to the cardiovascular, gastrointestinal, genitourinary, respiratory, neurovascular, neuroendocrine, and pupillomotor systems. Studies have reported a wide range of prevalence estimates for DAN in individuals with T1DM, ranging from 1 to 90%. While clinically manifest DAN is rare, subclinical DAN has been observed to develop within 2 years in patients with T1DM [29].

Certain researchers have proposed that CAN might advance silently before becoming clinically evident. Nevertheless, both clinically manifest autonomic neuropathy and subclinical forms, particularly CAN, pose a significant risk of morbidity and mortality in children with T1DM [1].

Symptoms indicative of cardiac autonomic neuropathy (CAN) encompass palpitations at rest, exercise intolerance, and signs suggestive of orthostatic hypotension (e.g., poor posture, fainting, dizziness, visual impairment, and syncope) [30].

Indeed, studies have demonstrated a relationship between poor metabolic control, older age, and longer duration of diabetes with autonomic dysfunction. There is evidence to suggest that increased autonomic dysfunction is often observed in patients with poor glycemic control.

In individuals with CAN, there is often a decrease in cardiac vagal regulation, which refers to the influence of the vagus nerve on the heart, and an increase in sympathetic cardiovascular markers. With a decrease in baroreflex sensitivity, the sympathetic system is stimulated, and tachycardia develops with its effect on the sinoatrial node. The duration of T1DM and impaired glycemic control (HbA1c > 8.5) over time have been associated with arterial stiffness and postural hypotension. In one study, we observed that compared to children without CAN, children with CAN had a longer duration of diabetes (more than 5 years), a significant number of diabetic complications, and worse glycemic control compared to those without CAN, but no differences were observed in age, gender, BMI, or blood pressure [30].

In our study, we enrolled asymptomatic patients with a mean age of 13.73 ± 1.96 years and a mean duration of diabetes of 8 ± 3.66 years. The one-year average HbA1c value was 11.34 ± 2.14%, ranging from 8.5 to 16.4%, indicating poor metabolic control according to the ISPAD 2022 consensus guidelines [21]. The patients enrolled in our study did not exhibit comorbidities and complications commonly associated with T1DM, such as hypertension, dyslipidemia, retinopathy, nephropathy, and neuropathy. In our study, we included patients who shared similar age, height, weight, and BMI. The specific focus of the study was on individuals at a high risk of developing CAN with poor metabolic control.

Our objective was to identify diagnostic tests capable of detecting CAN in asymptomatic patients within this cohort. All patients in the study underwent a comprehensive set of diagnostic assessments, including echocardiography, a 24-h rhythm Holter examination to assess HRV, a CPET, and a tilt table test.

In healthy individuals, the constant variation in intervals between heartbeats is a normal physiological occurrence. These periodic fluctuations in heart rate result from respiratory, thermoregulation, and baroreflex mechanisms. Vagal indices of heart rate variability tend to increase at night, while sympathetic indices show an increase during the day. Heart rate monitoring is a non-invasive technique used to illustrate autonomic neural dysfunction of the heart. A reduction in heart rate variability serves as a crucial indicator of the risk of sudden death and overall mortality. Parasympathetic and sympathetic autonomic dysfunctions have been reported at significantly higher frequencies in children with moderate and poor glycemic control. Increased adrenergic activity or decreased protective parasympathetic activity have been proved to cause diastolic dysfunction and fatal dysrhythmias, eventually increasing the mortality of T1DM as a complication. The findings from various studies indicate that the duration of diabetes exceeding 5 years, the presence of diabetes complications, and poor glycemic control are significantly associated with CAN in children with T1DM. However, no significant associations were observed with age, gender, or BMI [31, 32].

During the 24-h Holter examination of patients in our study group, it was observed that the average heart rate exceeded the average heart rate calculated based on age (Table 4). Subclinical CAN is prevalent in children and adolescents with T1DM. Parasympathetic and sympathetic autonomic dysfunctions have been reported at significantly higher frequencies in children with moderate and poor glycemic control [30]. Notably, there is a marked impact on parasympathetic nervous system dysfunction in comparison to sympathetic dysfunction.Chessa et al. [17] conducted a 24-h analysis of heart rate variability (HRV) in 50 asymptomatic patients with T1DM. Their findings revealed significant alterations in the square root of the mean square differences of successive RR intervals (r-MSSD), the percentage of differences between adjacent normal RR intervals > 50 ms (pNN50), and the abnormal high-frequency (HF) band of spectral analysis of HRV. Young et al. [33] observed a significant correlation between poor glycemic control and the duration of diabetes with nerve dysfunction. The authors also noted significant abnormalities in HRV among individuals with poor metabolic control.

In our study, HRV was assessed through 24-h rhythm Holter monitoring in our patient group. The findings revealed that the average heart rate in the patient group was significantly higher than that in the control group. Additionally, there was a decrease in SDNN, SDANNI, SDNNI, RMSSD, pNN50, Total Power, LF, HF, and VLF values. In our study, we observed a decrease in total power, a reduction in heart rate variability, a decline in both low-frequency (LF) and high-frequency (HF) components, and no change in the LF/HF ratio. These findings are indicative of tachycardia associated with heightened sympathetic activity. These parameters are of particular significance for the early detection of diabetic autonomic neuropathy. Once diabetic autonomic neuropathy findings manifest, the 5-year mortality rate reaches 50%. Hence, it becomes crucial to detect it during the subclinical period. Observing changes in heart rate provides us with an opportunity for early detection and intervention.

Tachycardia resulting from sympathetic activation is typically accompanied by a notable decrease in total power. The reduction in time domain parameters of heart rate variability (HRV) not only holds negative prognostic significance but also facilitates the identification of autonomic neuropathy before the manifestation of clinical signs. Under controlled conditions, a decrease in the absolute power of low-frequency (LF) and high-frequency (HF) components has been observed in diabetic patients without apparent autonomic neuropathy. In diabetic neuropathy, LF and HF decrease, but the LF/HF ratio remains unchanged. The inability to increase LF during standing suggests decreased baroreceptor sensitivity or impaired sympathetic response [6].

Children with T1DM exhibited significantly higher heart rate frequencies in response to the standing position (POTs), active standing (30:15 ratio), and Valsalva maneuver, indicating parasympathetic ANS dysfunction. Additionally, there were abnormalities in blood pressure responses to cold, pointing towards sympathetic ANS dysfunction in these individuals.Postural hypotension (PH) is characterized by a decrease in systolic blood pressure (SBP) ≤ 20 mmHg and/or a decrease in diastolic blood pressure (DBP) ≤ 10 mmHg on the Tilt table test. The tilt table test is one of the assessments that reveal sympathetic autonomic dysfunction in T1DM [34, 35]. The occurrence of postural hypotension associated with the duration of diabetes and poor glycemic control varies between 3–35% in adult patients. In contrast to adults, there are limited studies on postural hypotension in children with T1DM. In our study, postural hypotension was identified in 6 patients (20%) during the assessment of tilt table test analyses.

Tachycardia is considered the earliest sign of myocardial performance impairment or autonomic dysfunction. Given that the heart rate in our patient group is higher than in the control group, it is anticipated that there may be alterations in diastolic filling patterns. Ventricular functions were assessed using Doppler Tissue Imaging (DTI), a diagnostic method unaffected by heart rate variations, volume, and age [25]. It was observed that both systolic and diastolic periods were shortened due to the elevated heart rate. Findings consistent with diastolic dysfunction of the ventricles were identified, including increased A, DECT, PHT, and MPI values, as well as decreased E/A and E values. In our study, diastolic dysfunction with preserved systolic functions (EF) was noted in patients with poor metabolic control. Myocardial Performance Index (MPI) tends to increase in diabetic patients. This elevation in MPI is considered indicative of diastolic dysfunction, suggesting that MPI can be an effective tool in revealing early signs of impaired cardiac function in individuals with diabetes. Monitoring MPI can contribute to the assessment and management of diastolic dysfunction, offering insights into the cardiovascular impact of diabetes [36–39]. In addition to right ventricular tissue Doppler examinations, Pulmonary Valve (PW) Doppler examination was conducted. When right ventricular compliance decreases, the right ventricle operates as a passive conduit, leading to an anticipated increase in antegrade flow in the pulmonary artery during atrial systole. In our study, there is an observed increase in antegrade flow velocity (PA) and duration (PAT) in the pulmonary artery, consistent with right ventricular diastolic dysfunction.

In M-mode echocardiography examination, no significant differences were observed in heart dimensions and left ventricular systolic functions (EF, SF) measurements based on age. Given that our patients were asymptomatic, it is an expected finding that there was no change in the size of the heart chambers and that systolic functions were preserved.

In our study, we conducted exercise testing on our patients to assess the cardiopulmonary exercise response of individuals with type 1 diabetes and to evaluate the impact of glycemic control on these responses. In healthy children, it is typical for systolic blood pressure to increase with exercise. However, diastolic blood pressure tends to remain relatively unchanged, primarily due to vasodilation in the working skeletal muscles. This response is a normal physiological adaptation to the increased demand for oxygen and nutrients during physical activity. In the diabetic patient group, there is a lower cardiac output during exercise, and higher diastolic blood pressure is observed compared to the control group. Studies have reported an increase in both systolic and diastolic blood pressure during exercise in individuals with diabetes. Moreover, the rise in diastolic blood pressure has been associated with the duration of diabetes and poor diabetic control. Maximal exercise capacity, often measured in metabolic equivalents (METs), is considered one of the most crucial prognostic parameters obtained in exercise testing. It serves as a strong indicator of maximal oxygen consumption. In terms of the blood pressure response to exercise, it is generally expected that blood pressure will increase with the escalating treadmill workload. However, diastolic blood pressure typically remains relatively stable during exercise [40].

In our study, no significant differences were observed in T1DM patients who underwent exercise testing compared to the healthy group in terms of exercise duration, maximum exercise capacity (MET), maximum heart rate, or maximum systolic and diastolic pressure. These findings suggest that, based on the parameters assessed, there were no significant disparities in exercise performance and cardiovascular response between the two groups.

We acknowledge certain limitations in our study, firstly, the small sample size. To address this, longitudinal and prospective studies are essential for a more comprehensive understanding. Secondly, due to the cross-sectional design of our study, the temporal relationship between the appearance of signs of CAN and the onset of the disease remains unknown.

## Conclusions

Cardiovascular autonomic neuropathy is a common complication of T1DM, often associated with the patient’s age and inadequate glycemic control. Autonomic dysfunction, marked by reduced baroreceptor sensitivity, is linked to various impairments such as decreased ventricular diastolic functions, compromised respiratory functions, and diminished exercise capacity. Early detection of this autonomic disorder is crucial, and methods such as assessing heart rate variability and conducting active orthostatic tests play a significant role in its early diagnosis.

Recognizing and addressing cardiac autonomic dysfunction in its initial stages can be crucial in preventing the development of cardiovascular events and improving overall patient outcomes. Regular monitoring and proactive management of glycemic control are essential components of a comprehensive approach to mitigate the impact of cardiovascular autonomic neuropathy in individuals with type 1 diabetes.

## Data Availability

The datasets used and/or analysed during the current study are available from the corresponding author on reasonable request.
